# Machine Learning Model Based on Lipidomic Profile Information to Predict Sudden Infant Death Syndrome

**DOI:** 10.3390/healthcare10071303

**Published:** 2022-07-14

**Authors:** Karen E. Villagrana-Bañuelos, Carlos E. Galván-Tejada, Jorge I. Galván-Tejada, Hamurabi Gamboa-Rosales, José M. Celaya-Padilla, Manuel A. Soto-Murillo, Roberto Solís-Robles

**Affiliations:** Unidad Académica de Ingeniería Eléctrica, Universidad Autónoma de Zacatecas, Jardín Juárez 147, Centro, Zacatecas 98000, Mexico; kvillagrana@uaz.edu.mx (K.E.V.-B.); gatejo@uaz.edu.mx (J.I.G.-T.); hamurabigr@uaz.edu.mx (H.G.-R.); jose.celaya@uaz.edu.mx (J.M.C.-P.); alejandro.somu@uaz.edu.mx (M.A.S.-M.); rsolis@uaz.edu.mx (R.S.-R.)

**Keywords:** SIDS, lipidomic, metabolomic, glycerophospholipids, machine learning, biomarker

## Abstract

Sudden infant death syndrome (SIDS) represents the leading cause of death in under one year of age in developing countries. Even in our century, its etiology is not clear, and there is no biomarker that is discriminative enough to predict the risk of suffering from it. Therefore, in this work, taking a public dataset on the lipidomic profile of babies who died from this syndrome compared to a control group, a univariate analysis was performed using the Mann–Whitney *U* test, with the aim of identifying the characteristics that enable discriminating between both groups. Those characteristics with a *p*-value less than or equal to 0.05 were taken; once these characteristics were obtained, classification models were implemented (random forests (RF), logistic regression (LR), support vector machine (SVM) and naive Bayes (NB)). We used seventy percent of the data for model training, subjecting it to a cross-validation (k = 5) and later submitting to validation in a blind test with 30% of the remaining data, which allows simulating the scenario in real life—that is, with an unknown population for the model. The model with the best performance was RF, since in the blind test, it obtained an AUC of 0.9, specificity of 1, and sensitivity of 0.8. The proposed model provides the basis for the construction of a SIDS risk prediction computer tool, which will contribute to prevention, and proposes lines of research to deal with this pathology.

## 1. Introduction

Sudden infant death syndrome (SIDS) is the leading cause of infant mortality after the neonatal period in developed countries [1], and it is defined as death that remains unexplained, after having exhausted clinical and forensic investigations in children under one year of age [2]. Therefore, it is considered a diagnosis of exclusion; there are multiple theories that try to explain what causes this death without fully clarifying the physiopathogenesis of how or why it occurs. One of the most accepted theories is the triple risk theory, which implies that a baby is vulnerable, in a critical period of development, and there was a trigger. Where there are genetic factors that alter the central nervous system, cardiac channelopathies have been associated [3,4]; as well as inborn errors of metabolism; brainstem dysfunction and cardiorespiratory function; respiratory obstruction; infections; heat stress; and compression of vertebral arteries; among many other factors [5]. Taking this and modifiable risk factors into account, attempts to avoid these deaths have focused on prevention, especially safe sleep [1]; however, tools need to be investigated to help identify these patients. Despite the success obtained from the safe sleep campaigns, inquiry is required in order to obtain answers, which is why, at the moment, the realization of postmortem directed genetic tests (molecular autopsy) is recommended within the investigation of the cases [3].

Lipidomics and metabolomics have been described in the literature as optimal approaches to find differences in a particular physiological state. For example, a study of the metabolic profile in urine of preterm newborns of mothers with and without chorioamnioitis was carried out, where they found differences between the metabolites that were able to distinguish those born to mothers who suffered from this pathology or not [6]. Other authors [7] have worked with the metabolic profile in postmortem brains and identified changes in the urea as well as changes in carbohydrate and protein metabolism.

Metabolomics currently in disease research shows promise for biomarker discovery. Biomarkers are useful for predicting or identifying a risk of disease, diagnosing it, monitoring or prognosis [8]. Lipidomics is found within the metabolomics [9]; independently, it is dedicated to the study and characterization of the set of cellular lipids, the molecules with which they interact and their functions in the body, showing that the classic functions with which they are associated with lipids are those of structure and energy storage. However, technological advances have shown that there are different lipids in the human body suggesting the existence of functions not yet explored [10]. For example, in the detection of unexplored pathophysiological mechanisms for brain diseases [11], the potential value of metabolomics to study SIDS stands out. This study compares the profiles of the medulla oblongata in human brains of patients who died from SIDS with a control group, concluding that this type of study could lead to the antemortem identification of biomarkers [12]. Graham et al. directed a study of brain metabolomics in patients who died from SIDS compared to a control group, and they were able to identify possible biomarkers [13].

Omics technologies provide a large amount of data, which, when used in a univariate or multivariate manner, could contribute to the detection of the risk profile of the patient who dies from SIDS [14]. The large amount of data, which is produced with new technologies, benefits from the analysis with machine learning techniques. In the literature, it is described that through using machine learning (ML) techniques with characteristics between groups of cases and controls, it is possible to find biomarkers that help in medical diagnosis [15,16,17,18].

Decision making in medical diagnosis is a complicated process in which various factors are involved that can affect the certainty of doctors; for this reason, different ML techniques have been implemented to improve certainty in the diagnosis of diseases [19]. For example, Zoabi and collaborators used this type of technique in conjunction with clinical data to predict coronavirus disease [20]. Other researchers have implemented ML models for the prediction of cardiac arrhythmias [21]. ML has also been applied to cancer diagnosis [22], and it has also been recently applied to SIDS. For example, Blackburn and collaborators, taking into account infant mortality files and using unsupervised machine learning techniques, have identified groups of descendants of SIDS [23]. Galván-Tejada and collaborators; using clinical and short-chain fatty acid data, have also sought to aid in the diagnosis of SIDS [24]. However, it is necessary to continue investigating to identify the real cause of SIDS and effective models, which allow reducing more deaths from this pathology.

For this reason, in this work, the lipidomics of patients who died from SIDS are analyzed in comparison with control patients, and machine learning classification models are implemented in order to contribute to finding the risk profile and potential antemortem biomarkers that allow avoiding more deaths in the future.

## 2. Materials and Methods

**Figure 1:**
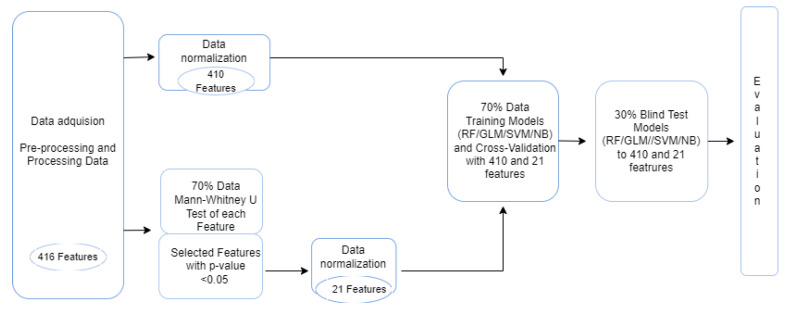
Flowchart of the steps that were followed for the development of the machine learning models, until their evaluation.

In this section, the materials and methods used are briefly described, and the steps that were carried out in the experimentation stage are visualized in Figure 1. All of the experimentation was developed in R (version 4.1.0) [25], which is a free software environment for graphics and statistical computing.

A public dataset available in the National Metabolomics Data Repository of the NIH Common Fund was used for this study. The dataset is named Lipidomics in sudden infant death syndrome, and it takes into account deceased patients diagnosed with SIDS (cases) and deceased patients from any other cause (controls) [26]. Lipid values were extracted from serum samples, and everything related to their processing regarding clinical analysis can be found in the “sample preparation” section where the public dataset was found.

### 2.1. Data Description

The information contained in the dataset “Lipidomics in sudden infant death syndrome” includes 6 characteristics of clinical information (post-conception age, postnatal age, gestational age, patient identification numbers, and the class or diagnosis—that is, case or control patient. The first includes patients who died and were diagnosed as due to SIDS and the second corresponds to the death of the patient from any other cause other than SIDS). The rest of the information is divided into two groups; the first corresponds to the negative mode analysis of the C18 ion, in which there are 132 characteristics (lipids), and there are 278 in the positive C18 ion. In total, there are 416 characteristics obtained postmortem in 33 patients, of which 23 correspond to cases and the rest are controls.

Each of them was grouped by the super class; for easy viewing, Table 1 shows the first column, which corresponds to the group, and the second column corresponds to the number of features in this group for the ion C18 negative mode analysis. Table 2 shows the same distribution of information, but it corresponds to the ion C18 positive mode analysis. So, joining both modes of analysis, we have 16 different groups of lipids.

### 2.2. Data Preprocessing

In this work, only the characteristics corresponding to lipids were included, uniting in a single data set both the mode analysis positive and negative C18 ion characteristics. So, 410 characteristics were used in total without including the class or diagnostic feature. We included 33 patients; 23 of them were classified as death by SIDS and the rest died from any other diagnosis, so they are considered control cases.

An exhaustive search was carried out to look for missing data, which were not found; therefore, there was no need to impute the data. The diagnostic variable was converted into binary values, representing 0 for control patients and 1 for SIDS cases.

### 2.3. Data Normalization

For the normalization of the data, the conversion of the values to *z*-scores was carried out, which represents how many standard deviations below or above the mean is the value to be evaluated from a reference population. This is exemplified in Equation (1), where *x* is the observed measure, mu is the population mean, and sigma is the population standard deviation [27].

(1) $$z=\frac{x-\;mu}{\sigma }$$

### 2.4. Classification Methods

Classification is one of the uses of supervised learning, which aims to predict class labels. In the case of medicine, with a binary classification, it seeks to identify the diagnosis: whether a patient is sick or healthy. Each of the implemented methods is described below and were developed in R [25], a free software environment for statistical computing and graphics.

Random forest (RF) [28] is based on multiple decision trees, so it has the advantage of decreasing the possibility of overfitting; each tree has a prediction of class, and finally, there is a mean to determine to which class each patient belongs: for example, if a patient is sick or not.

Each tree follows a series of logical decisions, similar to a flow chart, with decision nodes indicating a decision to be made about an attribute. Each branch indicates the decision options, which are assigned a predicted class. An important decision is to choose the best split, so that the ideal is that the partitions contain examples of a single class. If this happens, they are considered pure segments. One of the measures to measure this purity is entropy; the minimum value of 0 indicates that the sample is completely homogeneous, while 1 indicates the maximum amount of disorder. In the entropy Equation (2), for a given segment of data (*S*), the term *n* refers to the number of different class levels, and $P_{i}$ refers to the proportion of values falling into class level *i*. In simple terms, the total entropy resulting from a division is the sum of the entropy; each of the *n* partitions is weighted by the proportion of examples that fall in that partition ($w_{i}$) [29]. We used the package ’randomForest’ [30]. It is important to point out that all the functions mentioned in this work were used with the pre-established hyperparameters.

(2) $$\begin{array}{c} Entropy\left(S\right)=\sum_{i=1}^{n}w_{i}Entropy\left(P_{i}\right) \end{array}$$

Logistic regression (LR) [31] is used for binary classification, modeling the probability of belonging to one group or another. It transforms the value obtained with the linear regression (β + $\beta_{1}$X), using a function; the most used is the sigmoid function (Equation (3)), which will result in a value between 0 and 1. In other words, it is useful when you are interested in the impact of different explanatory variables on a binary response variable [32]. We used the funtion ${}^{‘}glm^{’}$ into the package ‘stats’ [33].

(3) $$\sigma \left(x\right)=\frac{1}{1+e^{-x}}$$

Support vector machine (SVM) is also useful for classification; it is considered a robust method, since it combines characteristics of the nearest neighbors and regression methods [29]. Its objective is to predict the new sample class, with the prior learning of the training. SVM uses a linear boundary called a hyperplane to divide data into groups of similar elements shown in Equation (4). Generally, as indicated by the class values, assuming you have two classes, the hyperplane, constructed, optimally separates, or whichever has the maximum distance (margin); a good separation between the classes will allow a correct classification [34].

(4) $$\bar{w}\cdot \bar{x}+b=0$$

However, in practice, some relationships between variables are not linear, so a kernel function can be applied to transform the features, assigning the data to a different dimensional space to achieve separation between classes. There are different types of kernels, such as linear, polynomial, or sigmoid; on this occasion, the radial kernel was used, also known as the radial basis function (RBF), which is popular for its similarity to the Gaussian distribution. It is represented by Equation (5) [35]. The ’e1071’ package was used with the ’svm’ function [36].

(5) $$K\left(x_{1},x_{2}\right)=exp\left(-\frac{{∥x_{1}-x_{2}∥}^{2}}{2\sigma^{2}}\right)$$

Naive Bayes (NB) is a method that searches to describe the probability of occurrence of an event. Taking into account the Bayes theorem [37], the probability of occurrence of an event A is sought; since event B has already occurred, it is also known as conditional probability, and it is exemplified in the following Equation (6). Thus, the probability is determined of a sample belonging to one class or another.

(6) $$P\left(A|B\right)=\frac{P(B|A)P(A)}{P(B)}=\frac{P(A⋂B)}{P(B)}$$

The naive Bayes algorithm makes two assumptions about the data: the first is that the data are equally important and that they are independent, which is unlikely to be the case in reality. So, the general formula would be:

(7) $$P\left(y_{i}|X_{1},X_{2}...,X_{n}\right)=P\left(X_{1}|y_{i}\right)P\left(X_{2}|y_{i}\right)...P\left(X_{n}|y_{i}\right)$$

Such assumptions have lent themselves to error speculation; however, it is said to work well, since it is not important to obtain a careful probability estimate provided that the predicted class values are true. For instance, if a disease classification model correctly identifies sick a patient, it does not matter whether it was 51% or 99% as long as the predicted class is correct [29]. The ’naivebayes’ function was used [36].

### 2.5. Feature Selection

The selection of the characteristics was carried out through a filtering method, which consists of applying different statistical techniques. In this case, considering that the sample size is small, according to the literature, the use of non-parametric tests is useful, which are used when the data do not present a Gaussian distribution. According to the literature, the Mann–Whitney *U* test [38] is useful when the normal or Gaussian distribution of the data cannot be identified, due to a small sample of the population, as is the case here. It takes into account the medians to know if there are differences between two independent populations [39], as in the case of patients who died from SIDS and control cases, according to each of the characteristics or lipids in this case.

The characteristics are ordered independently of the population to which they belong in ascending order to obtain the ranks. Subsequently, the Mann–Whitney *U* is calculated for each characteristic with Equations (8) and (9), where $n_{1}$ corresponds to the first sample population and $n_{2}$ corresponds to the second population, *R* represents the sum of the ranges of the population sample, respectively, obtaining the *p* value for 95% of statistical significance. The ’wilcox.test’ function was used [40].

(8) $$\begin{array}{c} U_{1}=n_{1}n_{2}+\frac{n_{1}\left(n_{1+1}\right)}{2}-R_{1} \end{array}$$

(9) $$\begin{array}{c} U_{2}=n_{1}n_{2}+\frac{n_{2}\left(n_{2+1}\right)}{2}-R_{2} \end{array}$$

### 2.6. Cross-Validation

**Figure 2:**
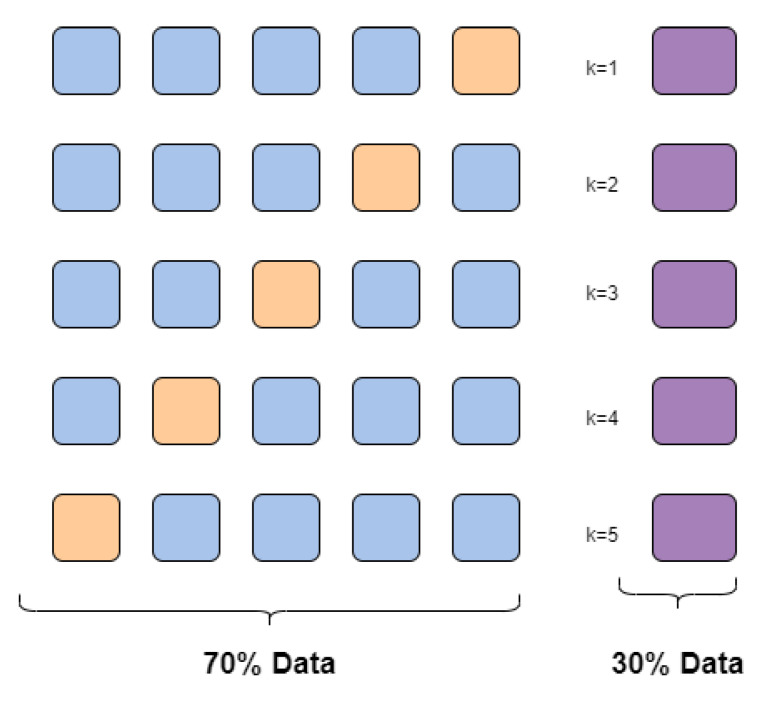
Exemplification of the cross-validation used in this work.

In this work, cross-validation was used, which is a statistical method that helps evaluate and compare learning algorithms. The main idea when using this method is that a part of the data set is hidden from the training model (the part shaded in orange in the Figure 2) in each iteration (*k*), and this repeats until at some point each of the folds created is used as a test. Then, we subject the model to a blind evaluation with the data that were kept out (the part shaded in purple in the Figure 2). This ensures that by subjecting the classification model to a new population, the algorithm will behave similarly to the test stage in the experimental phase, largely avoiding overfitting [41]. It was implemented with the function ’trainControl’ inside the ’caret’ package [42].

### 2.7. Metrics of Evaluation

There are two classification possibilities in this work: the SIDS patient or control case. Therefore, with the cases subjected to the model, a confusion matrix can be formed. To determine the true positive rate *(Sensitivity)*, that is, the probability that a sick subject has a positive test result, we use Equation (10), where *TP* refers to true positive cases (patients with disease classified as sick) and *FN* refers to false negatives (healthy patients classified as sick). The true negative rate *(Specificity)*, in other words, is the probability that a healthy subject will have a negative test result as represented by Equation (11), where *TN* are true negatives (healthy patients classified as healthy) and *FP* are false positives (sick patients classified as healthy); based on this, the ROC curve (receiver operation curve) is plotted, which represents the probability of the classifier to correctly determine a sample chosen at random, either positive or negative; and the area under the curve (AUC) was calculated. The higher the AUC, the better the model is at predicting 0 as 0 (healthy) and 1 as 1 (sick). This means that the higher the AUC, the better the model will be at distinguishing between patients with disease and without disease. That is, the model is excellent if it has an AUC of 1, which is interpreted as that the model has a good measure of separability. On the other hand, if the AUC is close to 0, it is the worst measure of separability: that is to say that instead of classifying 1 as 1, it does so as 0, and vice versa. The AUC of 0.5 represents that the model has no class separation capability, which is equivalent to making a random decision [43,44,45,46].

(10) $$Sensitivity=\frac{TP}{TP+FN}$$

(11) $$Specificity=\frac{TN}{TN+FP}$$

Another metric used was *accuracy*, which is a statistical measure of how well a binary classification test correctly identifies or excludes a condition. In other words, it is the proportion of true results among the total number of cases examined and is represented by the following Equation (12), where *TP* are true positives (sick patients identified as such), *TN* are true negatives (healthy patients identified as such), *FP* are false positives (healthy patients identified as sick) and *FN* are false negatives (sick patients identified as healthy) [47]. For this stage, the ’caret’ [48] and ’pROC’ [49] libraries were used.

(12) $$Accuracy=\frac{TP+TN}{TP+FP+FN+TN}$$

## 3. Results and Experimentation

This section presents the experiments carried out for the development of this work as well as the results obtained. As the first step, the set of lipidomic features (410 plus the class) were subjected to four different classification methods such as random forest (RF), logistic regression (RL), support vector machine (SVM), and naive Bayes (NB). These were used to predict 0 (control patient) or 1 (patient died from SIDS), using 70% of the data and cross-validation with *k*-folds (5). Subsequently, each of the trained models was submitted to evaluation with the remaining 30% of data, performing a blind test, and the evaluation metrics reported in Table 3 were obtained. Different rates for splitting the dataset are reported in the literature for the training and testing stages; however, it was observed that using the 70/30 rate on a small dataset ensures that the same proportion of cases and controls is maintained.

**Figure 3:**
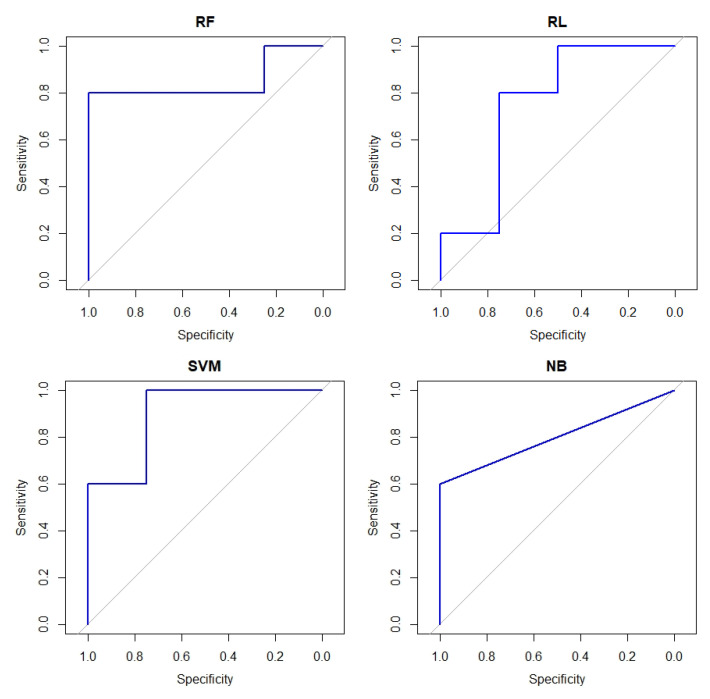
ROC curves of the four machine learning classification models, with the 21 selected features, blind test results.

Figure 3 shows the AUC obtained in the blind test of the models implemented with the 21 selected features. It is observed that regardless of the classification method used, all exceed 0.5 AUC, with the RF and SVM method achieving the best performance with a 0.9 AUC.

Then, a new experiment was carried out, using 70% of the data, which consisted of submitting each of the 410 characteristics to the Mann–Whitney *U* test, with which the *p*-value was obtained. Once this analysis was carried out, the characteristics with the *p*-value greater than 0.05 were selected, with a total of 21 characteristics, which are shown in Table 4. In this table, the name of the characteristic is shown in the first column, while the corresponding class to which it belongs, the chemical formula and finally the *p*-value obtained are observed on the right.

Each of the selected characteristics was subjected to normalization, using a *z*-score. With these selected characteristics plus the class or diagnosis, classification models were trained with the RF, RL, SVM and NB methods, dividing the data by 70 and 30 for cross-validation and blind testing, respectively. The same were evaluated in each stage, and the performance of each of the models is reported in Table 3.

## 4. Discussion

These results represent the first report describing a complete lipid profile analysis of cases and controls in SIDS.

A first experiment was carried out, which consisted of training the classification models, which will identify patients at risk of dying from SIDS compared to the control group (who died for any other reason), using four machine learning methods (RF, RL, SVM and NB), using 410 features (lipids values).

In the second experiment, using the statistical method of the Mann–Whitney *U* test, it was sought to know if it was possible to differentiate between the two groups with these features. We found that only 21 characteristics achieved this objective with a *p*-value of <0.05, that is, achieving statistical significance. In addition, each of the selected characteristics was subjected to normalization by means of *z*-score, and four classification models were subsequently implemented with the same automatic learning methods of the previous experiment in order to carry out the comparison.

Both experiments were subjected to cross-validation and exposed to evaluation with 30% of the data to perform a blind test, which means that the developed models had no prior knowledge of the new data to be evaluated, obtaining evaluation metrics for each one of them. In Table 3, the blind test stage of each one of the models is observed, with its two variants, with all the characteristics (410) and with the selected ones (21). It is clear that the models with 21 features present better performance compared to the models with 410 features. This is consistent with the literature [50,51], since a greater number of features does not ensure better performance of machine learning models, but on the contrary, they can be detrimental, as in this case.

According to the results, in the test stage, any of the four models (with 21 characteristics) present acceptable evaluations with an AUC greater than 0.75, specificity greater than 0.75, sensitivity greater than 0.6 and accuracy of 0.7777. However, the RF method stands out for having three of the four metrics evaluated with the best performance (AUC 0.9), which is followed by SVM (AUC 0.9), NB (AUC 0.8) and finally LR (AUC 0.75).

The NB model stands out by maintaining the same value in the accuracy and sensitivity metrics of the model with all and with the selected features. Based on the principle of independence of the characteristics for which this classifier is considered naive, it is difficult for such a statement to be true, since in the human body, metabolites and other body substances are in constant interaction. This model, according to the sensitivity, is the one with the worst performance regarding detecting the disease in sick subjects: it barely manages to overcome the random choice.

Such performances could be improved by balancing the data set. Different techniques allow for balancing data sets. For example, there are the resampling methods; within them are those of oversampling, which are responsible for increasing the minority class; on the other hand, those of subsampling reduce the minority class. However, they are controversial, since the use of subsampling techniques could lose valuable information, while oversampling techniques if the samples are few can lead to overfitting. For this reason, in this work, we decided not to implement them; however, it is interesting to know the behavior of the models when implementing these techniques and discuss them, which could be future work. Regarding the values close to the unit, as the sensitivity of 1, in the SVM model with 21 features, even using cross-validation techniques and the blind test would also benefit from testing the models with oversampling techniques or bigger data sets.

Of the lipids capable of discriminating between the two groups, 13 of the them correspond to the group of glycerophospholipids, of which 8 correspond to the group of glycerophosphocholines, 2 correspond to the group of glyphosphoethanolamines, 1 corresponds to the group of cardiolipins, 1 corresponds to the group of phosphatidylcholine, and 1 corresponds to the group of glycerophosphoglycerol. Of the remaining lipids, three belong to the group of sphingolipids and a subgroup of sphingomyelins, three belong to glycerolipids in subgroups of one triradyglyceroles and two diradylglycerols; and the remaining two belong to the sterol lipid group and sterol ester subgroup.

Hishikawa et al. [52] describe in their research the diversity of the functions of membrane glycerophospholipids in mammalian cells, which can be the constituents of cell membranes as well as precursors of cell signaling molecules and are part of lipoproteins and bile, among others. On the other hand, cone-shaped glycephospholipids with a small polar head (such as PE and CL), and/or bulky acyl chains, have important functions in membrane fusion, endocytosis, exocytosis, cytokinesis and in vesicle trafficking. That is to say that they are important in the transport of lipids. In addition, CL has been associated with problems of cellular respiration and energy production. It also stands out that lung surfactant, which is produced by type II pneumocytes, prevents lung collapse, and it is approximately 90% made up of lipids, and there are mainly dipalmitoyl-phosphatidylcholine and associated proteins in the remaining 10%. In other research, the role of glycerophospholipids in neural membranes is recognized, which is also relevant, since brain problems have been associated as causes of death from this syndrome [53,54,55]. This reinforces theories about pulmonary defects and at the same time opens possible lines of research since, when found in the results of this work, the ability to differentiate patients who died from SIDS from the control group, with glycerophospholipids, could lead to finding answers about the real origin of this pathology.

In the related literature, the work of Graham et al. [12,13] stands out, as they have used undirected metabolomics in their first work and directed metabolomics in their second to be able to identify babies who die of SIDS against those who do not, achieving AUC values of 1 and 0.92, respectively. However, they recognize some limitations in their work, such as that the cohort is not balanced, and that it is necessary to determine if their proposal is of clinical utility in blood. In contrast to the work that we present, the lipidomic profile was used, using a sample in peripheral blood, which could be useful for accessibility. It is relevant that our experimentation includes a validation or blind test with 30% of the data, which the trained model is totally unaware of. This allows reinforcing the performance of the model and visualizing the behavior it will have in real life, simulating that it is subjected to a different and unknown population.

## 5. Conclusions

The information presented regarding the lipids capable of discriminating between babies who died or not due to SIDS provides valuable information to the state of the art. To our knowledge, it is the first work that reports the lipidomic profile in babies who die from SIDS.

The 21 characteristics used for the predictive models are potential biomarkers that are capable of predicting together (multivariate model) if a patient is likely to die from this cause. It is encouraging because lines of research emerge aimed at obtaining answers about what really causes death, so that in the future, an accurate risk profile can be obtained to prevent more deaths.

There are a few studies that used metabolomic data from children who died of SIDS to predict susceptibility to SIDS death [12,13]. It is invasive to obtain tissue samples, but in contrast, this study has the potential to use lipids that were obtained in blood. The values obtained in the blind test with 21 characteristics were very good, achieving a maximum AUC of 0.9 with the RF model, which could be used as a prediction method, and a computer tool for detect potential cases, allowing the capture and follow-up of patients with this condition.

However, it presents some limitations, such as the imbalance of the data and the small size of the sample, which will be possibly favored by a larger cohort.

An exhortation is also required from the health institutions to carry out a follow-up in this age group in order to make databases that allow obtaining relevant information, preferably in a larger cohort. A second approach that would be interesting is to analyze the 21 selected characteristics in order to reduce them according to the definitions of biomarkers in the [56] literature. Some can be considered as potential biomarkers and be submitted to the evaluation phase, both analytical and clinical, in order to verify their relevance in living patients. In addition, the metabolic or lipidomic pathways can tentatively manage to discriminate between cases and controls, which could help clearly identify patients with a higher risk of dying from this cause.

## Figures and Tables

**Table 1:** Grouped features according to their super class; ION C18 negative mode analysis.

Group	Number of Features
Cardiolipins	6
Sphingolipids	1
Acids	16
Glycerophosphate	1
Phosphatylcholine	24
Phosphatylethalonamine	24
Phosphatidylglycerols	15
Phosphatidylinositols	12
Glycerophosphoserines	9
Lysophosphatidylethanolamine	8
Ether Phosphatidylethanolamines	16

**Table 2:** Grouped features according to their super class; ION C18 positive mode analysis.

Group	Number of Features
Cholesterol esters	12
Diacylglycerols	37
Monoradylglycerols	2
Phosphatylcholine	37
Phosphatylethalonamine	11
Sphingomyelins	43
Triacylglycerols	98
Lysophosphatidylcholines	25
Ether Phosphatidylethanolamines	4
Ether Phosphatidylcholines	9

**Table 3:** Evaluation metrics for each classifier of the standardized dataset.

Classification Method	Features	AUC	Accuracy	Sensitivity	Specificity
RF	410	0.2857	0.4444	0.5714	0
RF	21	**0.9000**	**0.8889**	0.8000	**1**
LR	410	0.4500	0.5555	0.4000	0.7500
LR	21	0.7500	0.7777	0.8000	0.7500
SVM	410	0.7000	0.7777	0.8000	0.7500
SVM	21	**0.9000**	0.8888	**1**	0.7500
NB	410	0.6750	0.6666	0.6000	0.7500
NB	21	0.8000	0.7777	0.6000	**1**

**Table 4:** Features selected by Mann–Whitney *U* test of the lipidomic profile in SIDS.

Features	Super Class	Main Class	Sub Class ^1^	Formula	*p*-Value
PC 40:7	Glycerophospholipids	Glycerophosphocholines	PC	C${}_{48}$H${}_{82}$NO${}_{8}$P	0.00420
PI 36:2	Glycerophospholipids	Glycerophosphocholines	PC	C${}_{44}$H${}_{84}$NO${}_{8}$P	0.00420
PE 35:0	Glycerophospholipids	Glycerophosphoethanolamines	PE	C${}_{40}$H${}_{80}$NO${}_{8}$P	0.01060
DG 34:1	Glycerolipids	Diradylglycerols	DAG	C${}_{37}$H${}_{70}$O${}_{5}$	0.01308
PC.38.7	Glycerophospholipids	Glycerophosphocholines	PC	C${}_{46}$H${}_{78}$NO${}_{8}$P	0.01602
PE 34:3	Glycerophospholipids	Glycerophosphoethanolamines	PE	C${}_{39}$H${}_{72}$NO${}_{8}$P	0.02355
TG 57:8	Glycerolipids	Triradylglycerols	TAG	C${}_{60}$H${}_{100}$O${}_{6}$	0.02355
CL 70:5	Glycerophospholipids	Cardiolipins	CL	C${}_{79}$H${}_{144}$O${}_{17}$P${}_{2}$	0.02355
SM 40:1	Sphingolipids	Sphingomyelins	SM	C${}_{45}$H${}_{91}$N${}_{2}$O${}_{6}$P	0.02826
PC 30:2	Glycerophospholipids	Glycerophosphocholines	PC	C${}_{38}$H${}_{72}$NO${}_{8}$P	0.02826
PC 32:3	Glycerophospholipids	Phosphatidylcholines	PC	C${}_{40}$H${}_{74}$NO${}_{8}$P	0.03372
SM 36:2	Sphingolipids	Sphingomyelins	SM	C${}_{41}$H${}_{81}$N${}_{2}$O${}_{6}$P	0.03372
PC 33:1	Glycerophospholipids	Glycerophosphocholines	PC	C${}_{41}$H${}_{80}$NO${}_{8}$P	0.03372
CE 18:2.	Sterol Lipids	Sterol esters	Chol	C${}_{45}$H${}_{76}$O${}_{2}$	0.03372
DG 36:2	Glycerolipids	Diradylglycerols	DAG	C${}_{39}$H${}_{72}$O${}_{5}$	0.03372
PC 32:1	Glycerophospholipids	Glycerophosphocholines	PC	C${}_{38}$H${}_{73}$O${}_{10}$P	0.03999
PG 36:3	Glycerophospholipids	Glycerophosphoglycerols	PG	C${}_{42}$H${}_{77}$O${}_{10}$P	0.04717
CE 22:6	Sterol Lipids	Sterol esters	Chol	C${}_{49}$H${}_{76}$O${}_{2}$	0.04717
PC 40:10	Glycerophospholipids	Glycerophosphocholines	PC	C${}_{50}$H${}_{80}$NO${}_{8}$P	0.04717
PC 42:7	Glycerophospholipids	Glycerophosphocholines	PC	C${}_{50}$H${}_{86}$NO${}_{8}$P	0.04717
SM.30.1	Sphingolipids	Sphingomyelins	SM	C${}_{35}$H${}_{71}$N${}_{2}$O${}_{6}$P	0.04717

^1^ PC—Phosphatidylcholines; PE—Phosphatidylethanolamines; DAG—Diacylglycerols; TAG—Triacylglycerols; CL—Cardiolipins; SM—Sphingomyelins; Chol—Cholesterol esters; PG—Phosphatidylglycerols.

## Data Availability

The data used to support the findings of this study are public. These were download from and are available on the NIH Common Fund National Metabolomics Data Repository (NMDR) website, Metabolomics Workbench, https://www.metabolomicsworkbench.org, (accessed on 22 February 2021), where it has been assigned project ID PR000475. The data can be accessed directly through its Project DOI:10.21228/M8NS47. The acquisition of the data was supported by the NIH grant, U2C-DK119886, but is not related to this manuscript, since they were taken directly from the aforementioned repository.
